# Electrical Instruments for Medical Use

**Published:** 1872-11

**Authors:** P. S. Hayes

**Affiliations:** 123 Calumet Avenue; Chicago


Article IV.—*Electrical Instruments for Medical Use.* By P. S. Hayes, M.D. (Read before the Chicago Society of Physicians and Surgeons, Sept. 16, 1872.)

The first applications of electricity as a remedial agent date from the discovery of the Leyden jar. Nollet and Boze appear to have been the first who thought of the application of electricity for the cure of disease; and soon the spark and electrical frictions became a universal panacea for all of the diseases that may attack the human organism.

The means employed for the generation and application of electricity for medical use are divided into stationary and portable apparatus. The stationary apparatus is subdivided into instruments for generating and collecting static, frictional, or franklinic electricity; instruments for generating galvanic, voltaic, constant, direct, continuous, interrupted continuous, or battery current; instruments for generating induced, faradaic, or electro-magnetic electricity; and instruments for applying electricity to the human subject. Electricity is not inherent in bodies, but is made manifest in them by various causes, among which are friction, pressure, chemical action, heat, and magnetism.

Thales, six centuries before Christ, discovered that amber, when excited by friction, possessed some of the phenomena of electricity; but it was not until near the close of the sixteenth century, that Dr. Gilbert, physician to Queen Elizabeth, showed that this property was not limited to amber, but that other substances, such as sulphur, wax, and glass, also possessed it.

Some of the properties of static electricity are as follows: It is generated primarily either by friction or pressure. It is of high tension, (which is the actual force of an electrical charge to break down any non-conducting substance between two terminating electrified planes), and weak in quantity, and, in consequence of this characteristic, it will leap a space, varying in distance according to the amount of electricity accumulated, and the state of the atmosphere, from a fraction of an inch to the largest space ever traversed by a flash of lightning.

Two bodies charged with the same electricity repel each other; two bodies charged with opposite electricities attract each other. The re-composition of positive and negative electricities, when at high tension, is accompanied by a spark or brush of light. It is capable of producing mechanical effects, such as the splitting of wood and perforation of glass. It will set on fire inflammable material and fuse metals. Its chemical effects are weak, when compared with those of galvanic electricity.

Static electricity is used comparatively little as a therapeutic agent. Duchenne is the strongest advocate of this form of electricity.

The length of this paper will not allow of an extended description of the machines for generating static electricity, but I will mention those generally used. The plate electrical machine has probably been used the most for generating static electricity for medical use. In 1865, Holtz, a German physicist, invented a machine which took by surprise the scientific world. This machine is known in this country as the Holtz Machine; but in Germany it is called the "Influenz-electrir-maschine." It generates electricity wholly by induction, or influence, and in amount unprecedented in the annals of electrical machines.

At the present time, the Holtz machine, with the improvements suggested by Poggendorff, is the one par-excellent for generating static electricity. The term induction, or influence, has been used, and it is but fitting that the term be defined in relation to static electricity. Ganot defines it as follows: "An insulated conductor, charged with either kind of electricity, acts on bodies in a natural state placed near it, in a manner analogous to that of the action of a magnet on soft iron, that is, it decomposes the neutral fluid, attracting the opposite, and repelling the like kinds of electricity."

Machines for generating static electricity are very uncertain in their action. For their perfect action they have to be thoroughly dry, and every part warmed, and it is necessary that the air be also dry, so that the electricity will not be diffused as soon as generated.

Galvanic electricity is generated by chemical action. It is of large quantity and of low tension. Its thermal effects are very great; with a proper battery, platinum, the most infusible of metals, may be melted. Of the light produced by a battery of six hundred elements, or cells, Despretz says, that "a single moment's exposure to the light is sufficient to produce very violent headache and pain in the eye, and the whole frame is affected as by a powerful sunstroke."

Its chemical effects are no less wonderful than those already mentioned; the molecules of water are torn asunder, and its elements freed from the invisible bond of chemical affinity. By means of its chemical action, Davy discovered the metals sodium and potassium. All of the electro-plating, electro-gilding and electro-typing, are done by this the galvanic electricity. This is the form of electricity used in telegraphing, and man has girdled the earth with iron as a road for this fleet messenger. The essential parts of a cell or element for generating galvanic electricity, are two different metals and an intervening fluid. Connected with each metal is a wire for conducting the electricity generated. The liquid acts chemically upon the metal which is called the electro-positive, more than on the other, the electro-negative. Positive electricity is generated on the electro-positive metal, and from this passes through the liquid to the electro-negative metal; the converse is true of the negative electricity; therefore the conductor connected with the electro-positive metal conducts negative, while that from the electro-negative metal conducts positive, electricity. The force which is produced by the difference in chemical action on the two metals, is termed the electro-motive force; depending on this difference of chemical action, a series is formed, which is termed the electro-motive series. Of the principal metals, zinc stands at the electro-positive, while platinum and graphite are at the electro-negative end of the series.

The current from a battery constructed on the plan just given, is found to be weakened by three principal causes. The first is, the decrease of the chemical action due to the weakening of the exciting fluid. The second is, local action, which is the production of small closed circuits in the active metal from the impurities it contains. The third is, the establishment of secondary currents, which are in direct opposition to the current first generated in the battery, and which are produced by gases collecting on the metallic plates, to which the term polarization of the plate is applied; this action, long continued, may result in the deposition of the electro-positive metal on the electro-negative plate.

In the various batteries found in the market the causes of the weakening of the current are obviated in some degree. The first cause can never be entirely overcome; and in all, with the exception, it may be, of the specific-gravity battery, this rule holds good, that a battery begins to run down as soon as it is set up. The specific-gravity battery requires but a few minutes attention each week to have a perfectly uniform current for months. The second cause may be obviated by having the electro-positive metal perfectly pure, or, if it be of zinc, by amalgamating it with mercury. The third cause may be prevented by putting the plates in different liquids which are separated from each other by a porous partition or cup of unglazed clay. In the Smee battery this is obviated by covering the negative or platinum plate with a deposit of finely divided platinum, which prevents the hydrogen from adhering to the plate.

The battery pre-eminent for medical use is the Hill battery, a modification of the specific-gravity battery. The advantages of this battery are its constancy; running for months with but little care; giving an even current of large quantity and small intensity—a current which is par-excellent for medical use; and costing but little to keep it in running order. This battery, as invented by Dr. E. A. Hill of this city, consists of a glass jar, in the bottom of which is placed a disk of sheet copper, which is the electro-negative plate; a gutta-percha covered wire, soldered to the under side of the copper plate, rises to the top of the jar, and is the positive pole. Suspended above the copper plate by a brass hanger is a disk of zinc, concave on its under surface, with a round aperture in the centre. This is the electro-positive plate. To the hanger is attached a binding screw, which forms the negative pole.

The body of the battery fluid is a solution of sulphate of zinc. Crystals of sulphate of copper are added from time to time through the central aperture in the zinc; these rest on the copper plate, and are gradually dissolved, forming a blue layer over the copper, of a solution of sulphate of copper. The two plates are each in a separate solution, and they are kept in these solutions as thoroughly as if they were separated by a porous partition or septum.

Eight years ago, my father, Justin Hayes, M.D., replaced the Daniels battery he had been using with the Hill battery; since which time he has constantly used it. Dr. Hammond, in his late work, entitled "Diseases of the Nervous System," states that he has a battery of this form consisting of thirty cells. Of the cells which form this battery he says that "they possess, in an eminent degree, the peculiar qualities that are essential for a galvanic battery for therapeutic purposes."

In a galvanic battery for medical use the positive plate of one cell should be connected with the negative plate of the next throughout the battery—the positive electrode being connected with the positive pole at one end, while the negative electrode is connected with the negative pole at the other end of the series forming the battery. Should it, however, be desirable to use only a part of the cells, one of the electrodes may be attached to the corresponding pole of an intermediate cell. This is facilitated by means of a switch or plugging board, or, as Dr. Hammond terms it, a "current selector," which is so arranged that any number of cells, from one to the full capacity of the battery, may be employed, at the option of the operator. To modify the current still more than can be done by the switch board, an instrument, termed a rheostat, is sometimes employed. The rheostat is constructed on the principle that a metallic wire resists the passage of an electrical current through it, proportioned directly as to its length and inversely as to its section. The unit of resistance generally employed in this country and England for graduating rheostats, is the Ohmad or British Association unit. In the manufacture of some rheostats, however, Sieman's unit, which is the ninety-five one-hundredth of an Ohmad, is employed. For medical use the rheostat should be so arranged that the resistance of any number of Ohmads, from 10 up to 2200, can be employed, as desired.

Dr. Neftel recommends that the rheostat be used in an accessory circuit. We use it in this manner, and find that we are thus enabled to treat either the eyes, ears, or any of the delicate organs or nerve-centres of the most sensitive person. The patient between the electrodes completes the main circuit, while the rheostat is placed in an accessory circuit, which it either completes itself or with the aid of a galvanometer which shows the strength and direction of the current and the resistance interposed by the rheostat. To illustrate the principle on which a rheostat so arranged works, let us suppose the resistance of the human body to the passage of an electrical current to be equal to 1000 Ohmads; now if there is no resistance interposed in the accessory circuit by the rheostat, there will 1000 times more electricity pass through the accessory circuit than through the circuit in which the person is placed; but if the resistance of 1000 Ohmads be interposed in the accessory circuit, there will just as much electricity pass through the one circuit as through the other.

The galvanometer is an instrument which may be made to fulfill three principal indications. It will inform you if you have a current at all, and if properly constructed you can approximately tell the quantity and intensity of the current. This you cannot tell exactly, as there is no uniform standard by which the galvanometer is made. Yet it is accurate enough for all therapeutic purposes.

The law which governs the manufacture of this instrument is as follows: If an electrical current be passed above or below a magnetic needle free to move on its axis, and which is in the magnetic meridian, the needle will be deflected, and tend to assume a position at right angles to the direction of the current; but as soon as the current is disconnected, the needle will again assume the direction of the magnetic meridian.

An instrument, termed by Dr. Hammond a "current modifier,"—which consists of a tube of glass or of some other non-conducting material, filled with water, closed at one end by a metallic disk connected with the battery, and in the other end is fixed a movable metallic piston, connected with a wire, which is either connected to an electrode or completes an accessory circuit,—has been used in place of the rheostat. When the piston is pushed against the metallic disk at the end of the tube, a continuous metallic circuit is established, but by withdrawing the piston the resistance of the water is interposed between it and the metallic disk, and the farther the piston is withdrawn the greater will be the resistance. When the "current modifier" is used in the galvanic circuit, the water in it begins immediately to be polarized, the metallic disk and piston acting as electrodes, oxygen collecting on the one, while hydrogen collects on the other electrode. The collecting gases soon accumulate in such quantities as to overcome in part the adhesive attraction which exists between them and the electrodes, and are continually being given off, thus constantly changing the surface of the electrodes, which are exposed to the water, and rendering the resistance irregular and uneven, and the gases which accumulate on the electrodes produce a current in the opposite direction. The polarization may be obviated by using a solution of the sulphate of zinc and a pure zinc disk and piston, but in this arrangement the one will be corroded while the other receives a deposit of zinc.

Between the patient and the battery in the metallic circuit there should be a commutator or current changer, by means of which the direction of the current may be changed instantly without disturbing the electrodes.

An apparatus called the current interrupter is sometimes of value in the diagnostic use of electricity. A simple form of this instrument consists of a spring in the metallic circuit, so arranged that the current can be made and broken by intermittent pressure on the spring. The current can thus be broken either as slowly or rapidly as the operator may desire.

Batteries for surgical use may be divided into those for galvanic cautery and those for electrolysis. Batteries for the galvanic cautery should consist of but few cells, with a large surface and considerable electro-motive power. Middeldorpff's apparatus is composed of four large cells of Grove's battery, each of which consists of a cylinder of zinc with a surface of 312 square inches, immersed in dilute sulphuric acid, and separated, by a porous cup containing nitric acid, from the platinum plate, which has a surface of 250 square inches.

In Grenet's apparatus the plates are of zinc and graphite, and the exciting fluid consists of one part of sulphuric acid, five parts of water, and one hundred grammes of the bichromate of potassium. There is, however, this objection to the apparatus, that it has to be continually oxygenated by blowing air into the solution by means of a pair of bellows.

For electrolysis any galvanic battery suitable for medical use may be successfully employed.

Induced electricity occupies a place intermediate between the galvanic and static electricities. Induced electricity may produce violent physiological, luminous, calorific and chemical effects, and can give rise to new induced currents. The intensity of the shock produced by the induced currents is comparable in its effect to electricity in a high state of tension, as is found in static electricity; but as the induced current acts on the galvanometer to deflect the needle, it is present not only in a state of tension, but also in a dynamic condition, in which it resembles galvanic electricity.

In 1831 Faraday discovered that a galvanic current is able, by induction or influence, to develop electrical currents in conducting wires. For example: let two wires be placed parallel to each other a short distance apart, one of the wires being attached to a battery and the other to a galvanometer; the instant that the battery circuit is completed, (the making of the circuit, as it is termed,) the needle of the galvanometer will be deflected; and by the direction the needle is deflected, it is ascertained that a current is developed in the wire which is in connection with the galvanometer, and that the direction of this current is opposite that of the galvanic current. This is the current on making, or the inverse induced current. Its duration is only instantaneous, as the needle immediately returns to zero. Now, if the galvanic circuit be broken, a current is induced in the wire connected with the galvanometer, which has the same direction as the galvanic current, and directly opposite in direction to the inverse induced current. This is the current on breaking, or the direct induced current. Faraday called these currents of induction. If, instead of the wires being parallel, they are insulated and coiled in the form of spirals or helices, the induced current will be increased. The helix which is in connection with the battery, is called the primary coil, and should be the smallest in diameter, so that it will fit into a cylindrical space in the centre of the outer or secondary coil. What has been said in regard to inducing a current in a wire insulated from the one completing the galvanic circuit, may also be applied with equal force to the one in the galvanic circuit. When this wire is coiled into a helix, the current, on making and breaking, is rendered more intense, which phenomenon Faraday refers to the inducing action which the current in each coil exerts upon the adjacent coils. These currents are known as the extra, or the so-called primary currents. The current generated upon closing the circuit is called the extra current on closing, or the inverse extra current, which is in a direction opposite that of the galvanic current. That generated upon opening is the extra current on opening, or the direct extra current, which has the same direction as the galvanic current, and which it strengthens. A magnet introduced into the centre of a spiral, induces, at the instant of introduction, a current in the spiral. Upon withdrawing the magnet, another current, opposite in direction to the first, is induced. These currents compare with the currents induced by the galvanic current. If, now, a core, which consists of a piece of soft iron, or, better, a number of soft iron wires varnished so as to be insulated from each other, be introduced into the primary coil, and the galvanic circuit be made, the core is instantly rendered a magnet, and produces the same effect as introducing a permanent magnet would. The instant the current is broken, the core ceases to be a magnet, and operates as though the magnet had been removed. This action of the core seconds the action of the galvanic current, and increases in intensity and quantity both the extra and the induced currents.

The requisites for an induction apparatus for medical use are: First, a core, which should either be a movable one or have a movable brass or copper cylindrical cover, for increasing or diminishing the current. Second, a primary coil, the wire of which should be large, not smaller than No. 16. From this coil branches should be taken, in order that the extra or primary currents can be used. Third, the secondary coil, the wire of which should be comparatively large, not smaller than No. 26. Fourth, a rheotome, or current interrupter, for interrupting the galvanic current. The usual form consists of an electro-magnet, a spring with an armature at one end, and a platinum pointed screw, which rests against the spring. Through this screw the electricity passes to the spring, and from thence around the electro-magnet, thereby rendering it magnetic. This magnet then attracts the armature, which, being drawn towards the magnet, breaks the connection; the electro-magnet then ceases to be magnetic, and at the same time to attract the armature, which, by the recoil inherent in the spring, is carried back to the point of starting, only to repeat again the operation just described. The rapidity of the vibrations may be governed in a measure by regulating the force by which the screw presses on the spring. The current should be broken regularly and evenly, for an uneven current is very disagreeable to the patient. Fifth, a commutator, as described under galvanic electricity. You may ask: What is the need of having a commutator, when the induced current is made up of two currents, opposite in direction to each other, a "to-and-fro current," as it is sometimes called, and each one deflects the galvanometer as much in one direction as the other one does in the opposite direction? Ganot gives the answer when he sums up the properties of induced currents, in the following words: "The direct induced current and the inverse induced current have been compared as to three of their actions: the violence of the shock, the deflection of the galvanometer, and the magnetizing action on steel bars. In these respects they differ greatly; they are about equal in their action on the galvanometer, *but while the shock of the direct current is very powerful, that of the inverse current is scarcely perceptible.* The direct current magnetizes to saturation, while the inverse current does not magnetize." Sixth, a rheostat, or "current modifier," for modifying the current still more than can be done by the core or its cover. The objection made to the "current modifier" under the galvanic current does not apply to it when used with the induced currents. Seventh, the battery for inducing the electricity. This should consist of one cell, with a large metallic surface of the plates, so that the inducing current may be of large quantity and small intensity, which characteristics it imparts in a measure to the induced currents. A Hill cell should present a zinc surface of at least 100 square inches.

The poles of the secondary as well as the extra current, are usually marked positive and negative.

The poles of the extra current are so marked from the corresponding poles of the galvanic current, which current forms one-third of the extra current, the other two-thirds being made up of formed by the direct extra, (which has the same direction as the galvanic), and the inverse extra currents.

In the secondary current, the poles of the direct induced current are the ones which give the distinction to the poles.

The electrode connected with the positive pole of an apparatus constructed on the principles just laid down, seems to exert a constringent action, while the negative relaxes. More attention should, I think, be given to the electrodes of the induction apparatus, and the position in which each is applied in the treatment of disease, than is usually done.

As a general rule the positive should be applied over the diseased or painful part. With this form of apparatus the biting intensity of the small machines sold for medical use is avoided, and the deep-seated structures and organs are reached with a power otherwise unattainable. The current is ofttimes felt deep in the muscles by the contractions it produces before the sentient nerves of the skin are acted on to any extent.

Silver is the best conductor of electricity known. If the conductivity of pure silver be represented by one hundred million, that of distilled water will be represented by the fraction one one-hundredth. A solution of the various salts are much better conductors than pure water. Water constitutes in the human subject between two-thirds and three-fourths of the entire weight of the body; and when we consider that all of the various salts found in the body are in solution in greater or less quantities in the various fluids found in it, we can readily see that, inside the epidermidal layer our bodies are better conductors than pure water. The epidermis contains less water than any other portion of the body, not excepting the teeth.

In proportion to the thickness of the epidermis is its resistance to electricity. If a dry electrode is applied to the skin, its effects are limited almost wholly to this tissue; but if the electrode be moistened with water, or, better, by water holding in solution one of the various salts, the resistance of the epidermis is in a great measure overcome, and the deeper-seated structures are reached.

If the body be placed in a tub constructed of non-conducting material filled with water, and an electrical current be made to pass through the water, the body will come in for a considerable share of the electricity. As electricity passes through the water it assumes the form of an ellipsoid, in consequence of the inferior conductivity of the water. In a body placed in the water between the electrodes no one point is effected more than a neighboring one; and thus all straining of separate organs is avoided. Should it be thought necessary to constringe, relax, or act upon any one portion of the human frame while in the water, the appropriate electrode may be placed on or over the part, while the opposite pole is connected with the electrodes of the tub. By this arrangement the electricity is radiated from the local part in every direction, so that no part is strained by the current.

An apparatus somewhat approximating the one just described, was invented and patented by the late Dr. Young, of Cleveland, several years ago, and was, as such things always are at first, very crude. The use of his patent was purchased by Dr. Justin Hayes, eleven years ago, who brought the apparatus to this city and used it in his practice as one of the means for the treatment of chronic diseases. This he found to be inefficient and cast it aside, and invented the one he now uses, which consists of a wood or soapstone tub, with stationary electrodes on the sides, and at the foot and head; each of these electrodes is connected with a key on a keyboard, and by means of the key can be made positive or negative or be thrown out of the circuit altogether. Thus any part or the whole of the body may be included in the circuit at once without touching the patient or tub.

Not only can the induced current be used in this apparatus, but also the galvanic can be connected to the key board and used. The temperature of the water used in the bath should not be less than 96° Fahrenheit, because electricity in a cool or cold bath stiffens the patient. The various salts and acids may be advantageously introduced into the bath. Should the sulphate of iron be used, its coloring property may be destroyed by the addition of sulphuric acid.

Of all electrodes for medical use, the hand is one of the best, but all hands are not good electrodes. The old zinc cylindrical electrodes are fast being superceded by those of a better pattern; as they were clumsy and inelegant, and did not meet the demands of the practitioner. The copper, acorn shaped, and silver plated electrode which was made for Dr. J. Hayes, and is at present used in his practice, fulfills the greatest number of indications of any electrode for general use.

The metallic brush is the form of electrode generally used when it is desirable to confine the action of the current to the cuticle. There are in the market electrodes for the urethra, vagina, uterus, pharynx and various other mucous surfaces situated near the external orifices of the mucous membrane. All of these electrodes may be purchased and used, but with what result? I had a patient say to me the other day, who had had one of these urethral electrodes introduced and the current applied to the prostate gland, that he "had rather die than have it done again, the operation was so painful."

Just within the horny ring that surrounds the orifices of the mucous membrane, the epithelial covering is so thin—in many places there being but a single layer—and the sensibility is so heightened, that anything but the normal secretions, excretions and ingesta produce irritation and pain, and the pain and irritation produced by the introduction of the electrode and the application of the electrical current is generally more than sufficient to balance the good produced, unless you wish to produce some profound revellent action, and even in this case the application of the current to the adjacent cutaneous surfaces in such a manner that the current may traverse the affected parts is often of much more value.

The electrodes for surgical use are those for cauterizing and those for electrolysis and electro-puncture. Electrodes for cauterizing consist of a fine wire of platinum, each end of which is connected with copper conductors, fixed in a suitable handle so that the current can be made and broken by the pressure of the finger. The resistance to the passage of the current is so great that the electrical force in part is converted into heat, which renders the wire incandescent. When it is necessary to cauterize a large surface, the platinum wire may be made to surround a porcelain button, which is rendered white hot and then quenched in the tissues to be cauterized. The electrodes for electrolysis and electro-puncture consist of one or more fine gold or gilt steel needles; these are sometimes termed acupuncture needles.

One of the most popular portable galvanic batteries is the Stohrer battery, which consists of a number of the Grenet cells described under batteries for surgical use, arranged as a compound battery. Each cell contains, when charged, about three ounces of fluid. The cells can be raised or lowered so that the plates can be immersed in the fluid when in use and be out of the fluid when not in use.

A year ago, Dr. Peck, at the instance of Dr. J. Hayes, had constructed by Bliss, Tillotson & Co., for his use, a portable instrument which combined the galvanic and induced currents in a more compact, complete and simple form than had ever been done before. The battery consists of fifteen Grenet cells, connected with a plugging board, which is so arranged that by the introducing or withdrawing the plugs some seventy different combinations can be made. For instance, all of the electro-positive and all of the electro-negative plates can be connected so as to form a battery of one cell; or, they may be combined so as to form a compound battery of from two to fifteen cells. The number of cells may be increased or diminished, one at a time. I have just had a machine made somewhat similar to Dr. Peck's. The battery is of the specific-gravity kind and consists of fifteen cells. Sawdust saturated with the solution is interposed between the plates. The united superficial area of the zinc plates is 100 square inches. The instrument contains a galvanometer and rheostat, and is the most perfect portable instrument that I have a knowledge of. Bliss, Tillotson & Co., manufacturers of and dealers in telegraphic supplies, 41 Third avenue, are the manufacturers of this instrument. They have the plans of it in their possession, from which they can at any time make an instrument to order.

In the form of machine called the magneto-electric the electricity is generated by induction from a permanent magnet. This kind of machine has nearly been discarded, as it requires a person to turn the crank to generate the electricity.

The induced electricity has been used as a remedial agent for less than thirty years. If one-half as many improvements in the construction of induction and galvanic apparatus, be made in the next thirty years, as have been made in the last thirty, our present instruments will soon be replaced by better ones.

The ancient weapons of warfare are superceded by breach-loading rifle and cannon, and the army that would win the battle must adopt the latest and best improvements. So should it be with the great army of physicians and surgeons, that they in their battle with disease and death, may be enabled to select from the archives of medical lore the latest and best means, and then use them with a perception and judgment that will be victorious.

123 Calumet Avenue.

---

***Calabar Beans for Constipation.***

Calabar beans have, until lately, been but little used except for contracting the pupil of the eye, and by Dr. Ogle, in cholera. Dr. Victor Subbotin, in the *Deutsch. Archiv. Klin. Med.*, proposes them as a remedy for atony of the bowel, observing that it has been found by Bauer, and confirmed by Besold and others, that this drug produces a cramp-like condition in organs that are supplied with involuntary muscular fibres.

He uses the following formula:

R. Ext. Sem. Physostig. Ven., - - grs. iv.
Glycerin. Pur., - - - - - drs. ij.
Solve. Sig. four drops four times daily.

He first administered this solution to a female who was much constipated from atony of the bowel. After the patient had used it twice, a swelling of the abdomen, caused by fæcal matter, disappeared permanently. Aloes and rhubarb had previously given temporary relief. The doctor has since treated successfully another case of the same nature, and one of chronic bronchial catarrh.—*Med. Record.*

